# Supplementary material for: Enhanced Heterologous Production of Glycosyltransferase UGT76G1 by Co-Expression of Endogenous prpD and malK in Escherichia coli and Its Transglycosylation Application in Production of Rebaudioside
Source: Int J Mol Sci. 2020 Aug 11;21(16):5752. doi: 10.3390/ijms21165752 (PMC7460871; doi:10.3390/ijms21165752)
Supplement: Supplementary file 1 [file ijms-21-05752-s001.pdf]

Enhanced heterologous production of glycosyltransferase UGT76G1 by co-expression of endogenous *prpD* and *malK* in *Escherichia coli* and its transglycosylation application in production of rebaudioside

Wenju Shu[1,2,†] Hongchen Zheng[1,2,3,†,*] Xiaoping Fu[2,3,†] Jie Zhen[2,3] Ming Tan[2,3] Jianyong Xu[2,3] Xingya Zhao[1,2] Shibin Yang[1,2] Hui Song[1,2,3,*] YanHe Ma[2,*]

[1]University of Chinese Academy of Sciences, Beijing 100049, China

[2]Industrial Enzymes National Engineering Laboratory, Tianjin Institute of Industrial Biotechnology, Chinese Academy of Sciences, Tianjin 300308, China

[3]Tianjin Key Laboratory for Industrial Biological Systems and Bioprocessing Engineering, Tianjin Institute of Industrial Biotechnology, Chinese Academy of Sciences, Tianjin 300308, China

[*]Corresponding authors. Address: No. 32 West 7th Avenue, Tianjin Airport Economic Area, Tianjin 300308, China. Tel: 086+022+84861934; Fax: 086+022+84861934. E-mail address: zheng_hc@tib.cas.cn (HongChen Zheng); song_h@tib.cas.cn (Hui Song); ma_yh@tib.cas.cn (YanHe Ma).

[†]Wenju Shu, Hongchen Zheng and Xiaoping Fu contributed equally to this work and are listed as co-first authors.

Table S1 Detail information of fusion partners in this work

Gene	Fusion expression partner	Protein sizes (kDa)	Organism
Fh8	Fasciola hepatica 8-kDa antigen	7.7	*F. hepatica*
MBP	Maltose-binding protein	42	*Escherichia coli*
Smt3	Small ubiquitin modified	11.3	*Synthetic*
DsbA	Disulphide isomerase I	23.1	*Escherichia coli*
DsbC	Disulphide isomerase	25.6	*Escherichia coli*

Table S2 Primers used to amplify the target genes in this work

Genes	Primer sequences
UGT76G1	5′ -ATGGAAAATAAAACGGAGACCACC -3′
	5′ -CAACGATGAAATGTAAGAAACCAAAGA -3′
Fh8	5′ -ATGCCGAGCGTTCAGGA -3′
	5′ -GCTGCTCAGAATGCTCAC-3′
MBP	5′ -ATGAAAATAAAAACAGGTGCACGCA-3′

	5′ -CTTGGTGATACGAGTCTGCG-3′
Smt3	5′ - ATGAGCGATAGCGAAGTGAA-3′
	5′ -ACCACCAATCTGTTCACG-3′
DsbA	5′ - ATGAAAAAGATTTGGCTGGCG-3′
	5′ -TTTTTTCTCGCTTAAGTATTTCACTGT-3′
DsbC	5′ -ATGAAGAAAGGTTTTATGTTGTTTACT-3′
	5′ -TTTACCGCTGGTCATTTTTTG-3′
prpD	5′ -ATGTCAGCTCAAATCAACAACATC-3′
	5′ -TTAAATGACGTACAGGTCGAG-3′
malK	5′ -ATGGCGAGCGTACAGCTG-3′
	5′ -TTAAACGCCCGGCTCCTTATG-3′

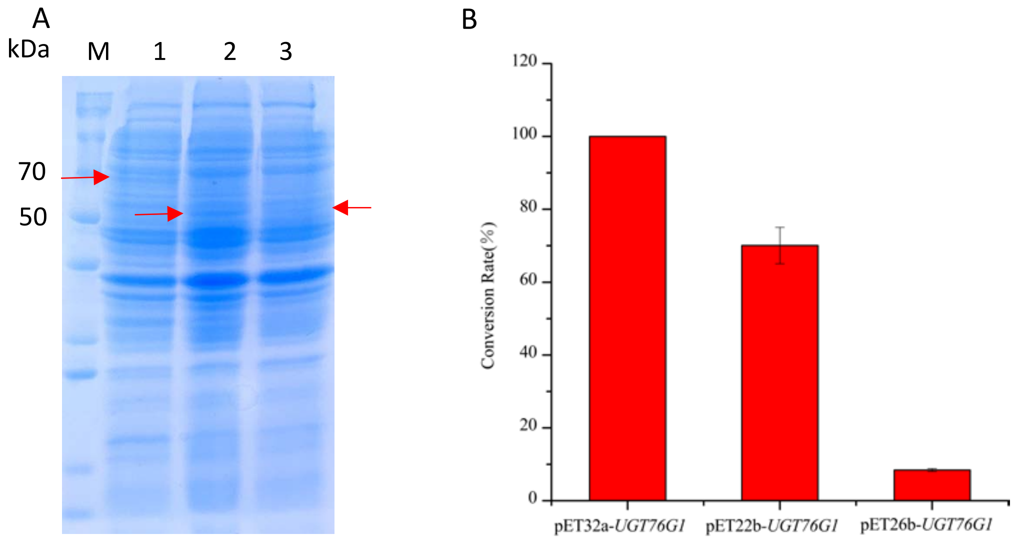

Figure S1 Expression of recombinant UGT76G1 under different plasmids in *E. coli*. A: SDS-PAGE of the whole cell lysates of different recombinant strains which harbouring different recombinant plasmids (1, pET32a-*UGT76G1*; 2, pET22b-*UGT76G1*; 3, pET26b-*UGT76G1*), M means marker of protein molecular weight; B: The different conversion rates from St to RA by the crude enzymes of the different recombinant strains in 12 h. Data are presented as mean±SD (n=3).

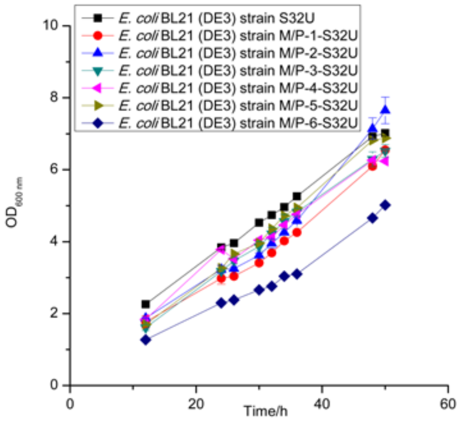

Figure S2 Growth profiles of the overexpression strains *E. coli* BL21 (DE3) M/P-(1-6) -S32U. The

detail information of these strains see Table 1. Data are presented as mean±SD (n=3).

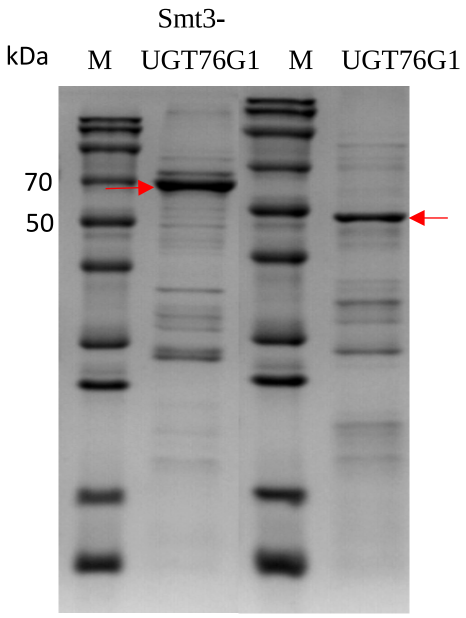

Figure S3 SDS-PAGE of the fusion enzyme Smt3-UGT76G1 and its tag deleted enzyme UGT76G1 by enterokinase hydrolysis
